# Supplementary material for: Rapid Sorting of Fucoxanthin-Producing Phaeodactylum tricornutum Mutants by Flow Cytometry
Source: Mar Drugs. 2021 Apr 17;19(4):228. doi: 10.3390/md19040228 (PMC8072577; doi:10.3390/md19040228)
Supplement: Supplementary file 1 [file marinedrugs-19-00228-s001.zip › marinedrugs-1161186-supplementary.pdf]

Supplementary Data

Rapid Sorting of Fucoxanthin-Producing *Phaeodactylum tricornutum* Mutants by Flow Cytometry

Yong Fan [1,2,3,4†], Xiao-Ting Ding [1,3,4†], Li-Juan Wang [1], Er-Ying Jiang [1,3,4], Phung Nghi Van [1,3,4] and Fu-Li Li [1,,3,4*]

[1] Key Laboratory of Biofuels, Shandong Provincial Key Laboratory of Synthetic Biology, Qingdao Institute of Bioenergy and Bioprocess Technology, Chinese Academy of Sciences, Qingdao 266101, China;
fanyong@qibebt.ac.cn (Y.F.); dingxt@qibebt.ac.cn (X.-T.D.); sunlibin@xiaoutech.com (L.-J.W.); jiangey@qibebt.ac.cn (E.-Y.J.); van@qibebt.ac.cn (P.N.V.)
[2] University of Chinese Academy of Sciences, Beijing 100049, China;
[3] Shandong Energy Institute, Qingdao 266101, China;
[4] Qingdao New Energy Shandong Laboratory, Qingdao 266101, China
* Correspondence: lifl@qibebt.ac.cn; Tel.: +86-532-80662655
† These authors have contributed equally to this work.

Supplementary Figure

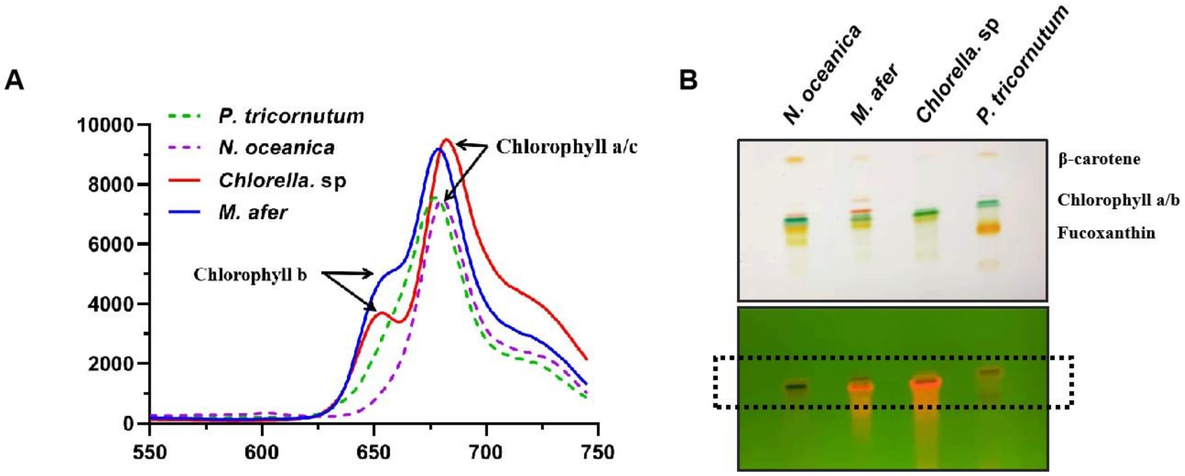

Figure S1. A. Fluorescence spectral scanning curves of four species of microalgae pigment extracts. All pigments were extracted using the same concentration of algal culture. The excitation wavelength is 488 nm. B. The pigment extracts of four microalgae were separated using TLC. The chlorophylls and carotenoids of the four microalgae were different. Blue light of 440-485 nm was used for fluorescence observation, and the samples containing chlorophyll a & b were shown strong visible red fluorescence, and the samples containing only chlorophyll a had only weak visible red fluorescence.

Supplementary data

A single-cell microalgae was screened from natural water in Qingdao near our laboratory (The microscopic pictures are shown below). We identified by sequencing 18S. the primers are 18S-F: 5’-AACCTGGTTATCCTGCCAGT-3’; 18S R 5’-ATCCTTCTGCAGGTTCACCTAC-3’. after sequencing and Blast, the result shown as below. We consider it a strain of *Chlorella* sp.

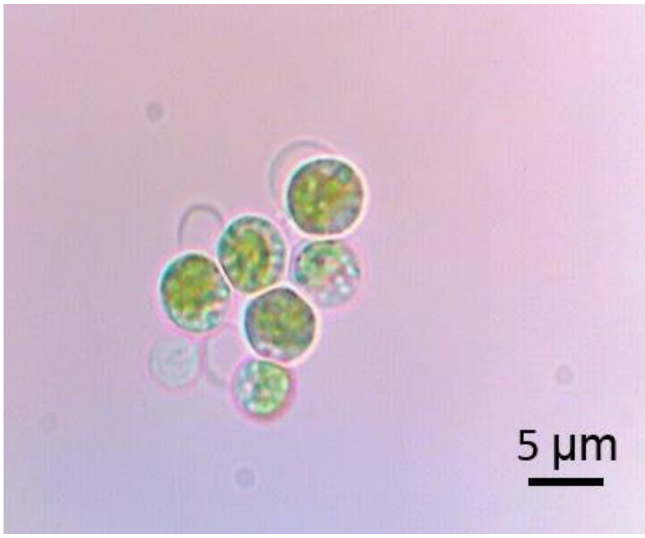

>Seq
AGTCAACGACTTCTCTTCCTCTAGGTGGGAGGGTTTAATGAACTTCTCGGCGGCCGAGAGCGGAGACCGCACCCGGTCGCCAATCC
GAACACTTCACCAGCACACCCAATCGGTAGGAGCGACGGGCGGTGTGTACAAAGGGCAGGGACGTAATCAACGCAAGCTGATGAC
TTGCGCTTACTAGGCATTCCTCGTTGAAGATTAATAATTGCAATAATCTATCCCCATCACGATGCAGTTTCAAAGATTACCCGGGCCTCT
CGGCCAAGGCTAGGCTCGTTGAATGCATCAGTGTAGCGCGCGTGCGGCCCAGAACATCTAAGGGCATCACAGACCTGTTATTGCCTC
ATGCTTCCATTGGCTAGTCGCCAATAGTCCCTCTAAGAAGTCCGCCGGCTGGCGAGCCAACCGTGACTATTTAGCAGGCTGAGGTCTC
GTTCGTTACCGGAATCAACCTGACAAGGCAACCCACCAACTAAGAACGGCCATGCACCACCACCCATAGAATCAAGAAAGAGCTCTC
AATCTGTCAATCCTCACTATGTCTGGACCTGGTAAGTTTTCCCGTGTTGAGTCAAATTAAGCCGCAGGCTCCACGCCTGGTGGTGCCC
TTCCGTCAATTCCTTTAAGTTTCAGCCTTGCGACCATACTCCCCCCGGAACCCAAAAACTTTGATTTCTCATAAGGTGCCGGCGGAGT
CATCGAAGAAACATCCGCCGATCCCTAGTCGGCATCGTTTATGGTTGAGACTAGGACGGTATCTAATCGTCTTCGAGCCCCCAACTTTC
GTTCTTGATTAATGAAAACATCCTTGGCAAATGCTTTCGCAGTAGTTCGTCTTTCATAAATCCAAGAATTTCACCTCTGACAATGAAATA
CGAATGCCCCCGACTGTCCCTCTTAATCATTACTCCGGTCCTACAGACCAACAGGATAGGCCAGAGTCCTATCGTGTTATTCCATGCTAA
TGTATTCAGAGCGTAGGCCTGCTTTGAACACTCTAATTTACTCAAAGTAACAGCGCCGACTCCGAGTCCCGGACAGTGAAGCCCAGG
AGCCCGTCCCCGGCAACAAGGTGGGCCCTGCCAGTGCACACCGAAACGGCGGACCGGCAGGCCCCACCCGAAATCCAACTACGAG
CTTTTTAACTGCAGCAACTTAAATATACGCTATTGGAGCTGGAATTACCGCGGCTGCTGGCACCAGACTTGCCCTCCAATTGATCCTCG
TTAAGGGGTTTAGATTGTACTCATTCCAATTACCAGACCTGAAAAGGCCCAGTATTGTTATTTATTGTCACTACCTCCCTGTGTCAGGAT
TGGGTAATTTGCGCGCCTGCTGCCTTCCTTGGATGTGGTAGCCGTTTCTCAGGCTCCCTCTCCGGAATCGAACCCTAATCCTCCGTCAC
CCGTTACCACCATGGTAGGCCTCTATCCTACCATCGAAAGTTGATAGGGCAGAAATTTGAATGAAACATCGCCGGCACGAGGCCATGC
GATTCGTGAAGTTATCATGATTCACCGCGAGTCGGGCAGAGCCCGGTCGGCCTTTTATCTAATAAATACGTCCCTTCCAGAAGTCGGG
ATTTACGCACGTATTAGCTCTAGATTTACTACGGGTATCCGAGTAGTAGGTACCATCAAATAAACTATAACTGATTTAATGAGCCATTCG
CAGTTTCACAGTATAAAGCAGTTTATACTTAGACATGCATGCTATACTAAAACT

Download GenBank Graphics ▼ Next ▲ Previous ◀De

Chlorella sp. ZJU0209 18S ribosomal RNA gene, partial sequence

Sequence ID: JX097061.1 Length: 1770 Number of Matches: 1

Range 1: 31 to 1741 GenBank Graphics ▼ Next Match ▲ Previous Match

Score	Expect	Identities	Gaps	Strand
3147 bits(1704)	0.0	1709/1711(99%)	1/1711(0%)	Plus/Minus

```
Query  6     ACGACTTCT-CTTCCTCTAGGTGGGAGGGTTTAATGAACTTCTCGGCGGCCGAGAGCGGA  6
             ||||||||| ||||||||||||||||||||||||||||||||||||||||||||||||||
Sbjct  1741  ACGACTTCTCCTTCCTCTAGGTGGGAGGGTTTAATGAACTTCTCGGCGGCCGAGAGCGGA  1

Query  65    GACCGCACCCGGTCGCCAATCCGAACACTTCACCAGCACACCCAATCGGTAGGAGCGACG  1
             ||||||||||||||||||||||||||||||||||||||||||||||||||||||||||||
Sbjct  1681  GACCGCACCCGGTCGCCAATCCGAACACTTCACCAGCACACCCAATCGGTAGGAGCGACG  1

Query  125   GGCGGTGTGTACAAAGGGCAGGGACGTAATCAACGCAAGCTGATGACTTGCGCTTACTAG  1
             ||||||||||||||||||||||||||||||||||||||||||||||||||||||||||||
Sbjct  1621  GGCGGTGTGTACAAAGGGCAGGGACGTAATCAACGCAAGCTGATGACTTGCGCTTACTAG  1

Query  185   GCATTCCTCGTTGAAGATTAATAATTGCAATAATCTATCCCCATCACGATGCAGTTTCAA  2
             ||||||||||||||||||||||||||||||||||||||||||||||||||||||||||||
Sbjct  1561  GCATTCCTCGTTGAAGATTAATAATTGCAATAATCTATCCCCATCACGATGCAGTTTCAA  1

Query  245   AGATTACCCGGGCCTCTCGGCCAAGGCTAGGCTCGTTGAATGCATCAGTGTAGCGCGCGT  3
             ||||||||||||||||||||||||||||||||||||||||||||||||||||||||||||
Sbjct  1501  AGATTACCCGGGCCTCTCGGCCAAGGCTAGGCTCGTTGAATGCATCAGTGTAGCGCGCGT  1

Query  305   GCGGCCCAGAACATCTAAGGGCATCACAGACCTGTTATTGCCTCATGCTTCCATTGGCTA  3
             ||||||||||||||||||||||||||||||||||||||||||||||||||||||||||||
Sbjct  1441  GCGGCCCAGAACATCTAAGGGCATCACAGACCTGTTATTGCCTCATGCTTCCATTGGCTA  1

Query  365   GTCGCCAATAGTCCCTCTAAGAAGTCCGCCGGCTGGCGAGCCAACCGTGACTATTTAGCA  4
             ||||||||||||||||||||||||||||||||||||||||||||||||||||||||||||
Sbjct  1381  GTCGCCAATAGTCCCTCTAAGAAGTCCGCCGGCTGGCGAGCCAACCGTGACTATTTAGCA  1

Query  425   GGCTGAGGTCTCGTTCGTTACCGGAATCAACCTGACAAGGCAACCCACCAACTAAGAACG  4
             ||||||||||||||||||||||||||||||||||||||||||||||||||||||||||||
Sbjct  1321  GGCTGAGGTCTCGTTCGTTACCGGAATCAACCTGACAAGGCAACCCACCAACTAAGAACG  1

Query  485   GCCATGCACCACCACCCATAGAATCAAGAAAGAGCTCTCAATCTGTCAATCCTCACTATG  5
             ||||||||||||||||||||||||||||||||||||||||||||||||||||||||||||
Sbjct  1261  GCCATGCACCACCACCCATAGAATCAAGAAAGAGCTCTCAATCTGTCAATCCTCACTATG  1

Query  545   TCTGGACCTGGTAAGTTTTCCCGTGTTGAGTCAAATTAAGCCGCAGGCTCCACGCCTGGT  6
             ||||||||||||||||||||||||||||||||||||||||||||||||||||||||||||
Sbjct  1201  TCTGGACCTGGTAAGTTTTCCCGTGTTGAGTCAAATTAAGCCGCAGGCTCCACGCCTGGT  1

Query  605   GGTGCCCTTCCGTCAATTCCTTTAAGTTTCAGCCTTGCGACCATACTCCCCCCGGAACCC  6
             ||||||| ||||||||||||||||||||||||||||||||||||||||||||||||||||
Sbjct  1141  GGTGCCCCTCCGTCAATTCCTTTAAGTTTCAGCCTTGCGACCATACTCCCCCCGGAACCC  1

Query  665   AAAAACTTTGATTTCTCATAAGGTGCCGGCGGAGTCATCGAAGAAACATCCGCCGATCCC  7
             ||||||||||||||||||||||||||||||||||||||||||||||||||||||||||||
Sbjct  1081  AAAAACTTTGATTTCTCATAAGGTGCCGGCGGAGTCATCGAAGAAACATCCGCCGATCCC  1

Query  725   TAGTCGGCATCGTTTATGGTTGAGACTAGGACGGTATCTAATCGTCTTCGAGCCCCCAAC  7
             ||||||||||||||||||||||||||||||||||||||||||||||||||||||||||||
Sbjct  1021  TAGTCGGCATCGTTTATGGTTGAGACTAGGACGGTATCTAATCGTCTTCGAGCCCCCAAC  9

Query  785   TTTCGTTCTTGATTAATGAAAACATCCTTGGCAAATGCTTTCGCAGTAGTTCGTCTTTCA  8
             ||||||||||||||||||||||||||||||||||||||||||||||||||||||||||||
Sbjct  961   TTTCGTTCTTGATTAATGAAAACATCCTTGGCAAATGCTTTCGCAGTAGTTCGTCTTTCA  9

Query  845   TAAATCCAAGAATTTCACCTCTGACAATGAAATACGAATGCCCCCGACTGTCCCTCTTAA  9
             ||||||||||||||||||||||||||||||||||||||||||||||||||||||||||||
Sbjct  901   TAAATCCAAGAATTTCACCTCTGACAATGAAATACGAATGCCCCCGACTGTCCCTCTTAA  8

Query  905   TCATTACTCCGGTCCTACAGACCAACAGGATAGGCCAGAGTCCTATCGTGTTATTCCATG  9
             ||||||||||||||||||||||||||||||||||||||||||||||||||||||||||||
Sbjct  841   TCATTACTCCGGTCCTACAGACCAACAGGATAGGCCAGAGTCCTATCGTGTTATTCCATG  7

Query  965   CTAATGTATTCAGAGCGTAGGCCTGCTTTGAACACTCTAATTTACTCAAAGTAACAGCGC  1
             ||||||||||||||||||||||||||||||||||||||||||||||||||||||||||||
Sbjct  781   CTAATGTATTCAGAGCGTAGGCCTGCTTTGAACACTCTAATTTACTCAAAGTAACAGCGC  7

Query  1025  CGACTCCGAGTCCCGGACAGTGAAGCCCAGGAGCCCGTCCCCGGCAACAAGGTGGGCCCT  1
             ||||||||||||||||||||||||||||||||||||||||||||||||||||||||||||
Sbjct  721   CGACTCCGAGTCCCGGACAGTGAAGCCCAGGAGCCCGTCCCCGGCAACAAGGTGGGCCCT  6

Query  1085  GCCAGTGCACACCGAAACGGCGGACCGGCAGGCCCCACCCGAAATCCAACTACGAGCTTT  1
             ||||||||||||||||||||||||||||||||||||||||||||||||||||||||||||
Sbjct  661   GCCAGTGCACACCGAAACGGCGGACCGGCAGGCCCCACCCGAAATCCAACTACGAGCTTT  6

Query  1145  TTAACTGCAGCAACTTAAATATACGCTATTGGAGCTGGAATTACCGCGGCTGCTGGCACC  1
             ||||||||||||||||||||||||||||||||||||||||||||||||||||||||||||
Sbjct  601   TTAACTGCAGCAACTTAAATATACGCTATTGGAGCTGGAATTACCGCGGCTGCTGGCACC  5

Query  1205  AGACTTGCCCTCCAATTGATCCTCGTTAAGGGGTTTAGATTGTACTCATTCCAATTACCA  1
             ||||||||||||||||||||||||||||||||||||||||||||||||||||||||||||
Sbjct  541   AGACTTGCCCTCCAATTGATCCTCGTTAAGGGGTTTAGATTGTACTCATTCCAATTACCA  4

Query  1265  GACCTGAAAAGGCCCAGTATTGTTATTTATTGTCACTACCTCCCTGTGTCAGGATTGGGT  1
             ||||||||||||||||||||||||||||||||||||||||||||||||||||||||||||
Sbjct  481   GACCTGAAAAGGCCCAGTATTGTTATTTATTGTCACTACCTCCCTGTGTCAGGATTGGGT  4

Query  1325  AATTTGCGCGCCTGCTGCCTTCCTTGGATGTGGTAGCCGTTTCTCAGGCTCCCTCTCCGG  1
             ||||||||||||||||||||||||||||||||||||||||||||||||||||||||||||
Sbjct  421   AATTTGCGCGCCTGCTGCCTTCCTTGGATGTGGTAGCCGTTTCTCAGGCTCCCTCTCCGG  3

Query  1385  AATCGAACCCTAATCCTCCGTCACCCGTTACCACCATGGTAGGCCTCTATCCTACCATCG  1
             ||||||||||||||||||||||||||||||||||||||||||||||||||||||||||||
Sbjct  361   AATCGAACCCTAATCCTCCGTCACCCGTTACCACCATGGTAGGCCTCTATCCTACCATCG  3

Query  1445  AAAGTTGATAGGGCAGAAATTTGAATGAAACATCGCCGGCACGAGGCCATGCGATTCGTG  1
             ||||||||||||||||||||||||||||||||||||||||||||||||||||||||||||
Sbjct  301   AAAGTTGATAGGGCAGAAATTTGAATGAAACATCGCCGGCACGAGGCCATGCGATTCGTG  2

Query  1505  AAGTTATCATGATTCACCGCGAGTCGGGCAGAGCCCGGTCGGCCTTTTATCTAATAAATA  1
             ||||||||||||||||||||||||||||||||||||||||||||||||||||||||||||
Sbjct  241   AAGTTATCATGATTCACCGCGAGTCGGGCAGAGCCCGGTCGGCCTTTTATCTAATAAATA  1
```
